# MEGADOCK 4.0: an ultra–high-performance protein–protein docking software for heterogeneous supercomputers

**DOI:** 10.1093/bioinformatics/btu532

**Published:** 2014-08-06

**Authors:** Masahito Ohue, Takehiro Shimoda, Shuji Suzuki, Yuri Matsuzaki, Takashi Ishida, Yutaka Akiyama

**Affiliations:** ^1^Department of Computer Science, Graduate School of Information Science and Engineering, Tokyo Institute of Technology, 2-12-1 W8-76, Ookayama, Meguro-ku, Tokyo 152-8550, Japan, ^2^Japan Society for the Promotion of Science (JSPS) and ^3^Education Academy of Computational Life Sciences (ACLS), Tokyo Institute of Technology, 2-12-1 W8-93, Ookayama, Meguro-ku, Tokyo 152-8550, Japan

## Abstract

**Summary:** The application of protein–protein docking in large-scale interactome analysis is a major challenge in structural bioinformatics and requires huge computing resources. In this work, we present MEGADOCK 4.0, an FFT-based docking software that makes extensive use of recent heterogeneous supercomputers and shows powerful, scalable performance of >97% strong scaling.

**Availability and Implementation:** MEGADOCK 4.0 is written in C++ with OpenMPI and NVIDIA CUDA 5.0 (or later) and is freely available to all academic and non-profit users at: http://www.bi.cs.titech.ac.jp/megadock.

**Contact:** akiyama@cs.titech.ac.jp

**Supplementary information:** Supplementary data are available at *Bioinformatics* online

## 1 INTRODUCTION

Protein–protein interactions can provide valuable insights for understanding the principles of biological systems and for elucidating causes of incurable diseases. Although many structures of interacting proteins have been determined by X-ray crystallography and Nuclear Magnetic Resonance spectroscopy, the structures of many protein complexes have still not been determined experimentally because of cost and technical limitations. Protein–protein docking, a computational method for predicting the structure of a protein complex from known component structures, is a powerful approach that facilitates the discovery of otherwise unattainable protein complex structures.

A number of fast Fourier transform (FFT)-based rigid-body initial protein–protein docking tools have been developed for predicting protein complex structures (Cheng *et al.*, 2007; Pierce *et al.*, 2011; Ritchie and Venkatraman, 2010). However, faster docking tools are still required to perform large-scale interactome predictions. Some applications also require a huge number of dockings, such as ensemble docking techniques using multiple conformations for flexible docking (Grünberg *et al.*, 2004; Król *et al.*, 2007), cross-docking for identification of protein interaction partners (Lopes *et al.*, 2013; Matsuzaki *et al.*, 2009; Wass *et al.*, 2011; Zhang *et al.*, 2014) and multiple docking (Karaca and Bonvin, 2011). To achieve these large-scale analyses, use of the supercomputing environment has become absolutely necessary.

On the other hand, 35% of computing performance of supercomputers ranked in top500.org (June 2014) is currently achieved by hardware accelerators, such as graphics processing units (GPUs), and this percentage is increasing. Therefore, tools that can be used with such ‘heterogeneous’ supercomputers are necessary. While some docking tools are accelerated by GPUs on a node (Ritchie and Venkatraman, 2010; Sukhwani and Herbordt, 2009), ‘heterogeneous’ supercomputers, which have massive numbers of nodes including multiple CPU cores and GPU cards, have not yet been used for acceleration of docking tool performance.

Here, we present ultra–high-performance docking software, ‘MEGADOCK 4.0’, which makes extensive use of supercomputers equipped with GPUs.

## 2 IMPLEMENTATION

### 2.1 MEGADOCK scheme

MEGADOCK uses a Katchalski-Katzir algorithm known as a traditional FFT-based rigid-docking scheme (Katchalski-Katzir *et al.*, 1992). Its original scoring function, based on shape complementarity, electrostatics and desolvation free energy, is calculated by only one correlation function (Ohue *et al.*, 2012, 2014). This is advantageous for faster calculation because multiple correlation functions and thus multiple FFT calculations are used to evaluate multiple effects in previous methods (Kozakov *et al.*, 2006; Pierce *et al.*, 2011). (see Supplementary Text S1 for details)

### 2.2 GPU implementation

MEGADOCK has been implemented on multiple GPUs using the CUDA library (Shimoda *et al.*, 2013). A previous study (Sukhwani and Herbordt, 2009) mapped only FFT processes onto a GPU, and its implementation could not use multiple GPUs. We mapped the whole docking process (voxelization, ligand rotation, FFTs and finding solutions) onto GPUs, and our implementation was able to use multiple GPUs and CPU cores (Shimoda *et al.*, 2013).

### 2.3 Hybrid CUDA, MPI and OpenMP parallelization

For extensive execution of docking jobs, an implementation that can be performed among many computing nodes is required. We previously parallelized the calculation of each docking processes using MPI and OpenMP with the master/worker model (Matsuzaki *et al.*, 2013). On cluster computers, a master process acquires a list of protein pairs and distributes the docking jobs to worker processes on available nodes. This implementation guarantees fault tolerance in that the master process surveys all docking jobs.

The proposed software, MEGADOCK 4.0, is implemented by hybrid CUDA, MPI and OpenMP parallelization. Reducing the usage of memory space is important with systems that have many CPU cores, multiple GPUs per node and relatively little memory (e.g. there is only 6 GB memory on an NVIDIA Tesla K20X GPU). We assigned one docking job to each node and then distributed the calculations of ligand rotation by thread parallelization with CPU cores and GPUs. This implementation model manages one node as the master and the other nodes as workers. The master node distributes the docking jobs to worker nodes, and a worker node executes distributed docking jobs with multiple GPUs by CUDA and all CPU cores by OpenMP thread parallelization. This implementation also guarantees fault tolerance similar to the CPU version.

## 3 RESULTS AND DISCUSSION

To check the performance of MEGADOCK 4.0, we used the ZLAB benchmark 4.0 dataset (Hwang *et al.*, 2010). Speed measurement experiments were conducted on the TSUBAME 2.5 supercomputing system (Tokyo Institute of Technology, Japan). We used its ‘thin nodes’ with a reservation service of exclusive use (up to 420 nodes). Each ‘thin’ node contained two Intel Xeon X5670 (six cores, 2.93 GHz) and three NVIDIA Tesla K20X (GK110) GPUs. The specifications of the environment are shown in Supplementary Text S2 and Table S1.

**Fig. 1.**
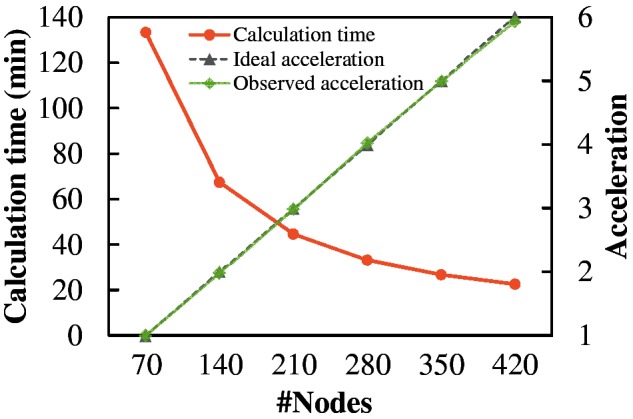
Calculation time and acceleration by parallelization among nodes on 30 976 docking jobs

Figure 1 shows the average of five measurements of computation time and the parallel scalability of MEGADOCK 4.0 on 30 976 protein pairs from combinations between 176 receptors and 176 ligands, assuming a cross-docking study. The observed calculation acceleration was close to ideal. Strong scaling values from 35 nodes were >97% for all numbers of nodes measured here (Supplementary Table S2). Notably, a high scalability (98%) was obtained with the largest number of nodes (420 nodes).

We also measured docking time on a half million and a million protein pairs for simulation of large-scale interactome analyses using averaged-sized proteins (FFT size of 108, see Supplementary Table S3). In this simulation, a half million docking jobs required 5.71 h, while a million jobs required 11.51 h. The epidermal growth factor receptor-related pathway, which we are studying in non–small-cell lung cancer, required approximately a quarter million dockings. This analysis could be completed in only 3 h with MEGADOCK 4.0 using 420 nodes, whereas solving the same problem requires several days with an older version of MEGADOCK.

## 4 CONCLUSIONS

MEGADOCK 4.0 is a docking software for heterogeneous supercomputing environments and shows excellent scalability. Heterogeneous supercomputers equipped with hardware accelerators, such GPUs, will become common in the future. Fully using such computers is crucial for bioinformatics research, which must analyze massive amounts of data. MEGADOCK 4.0 can serve as a tool to promote analysis of the whole interactome within a reasonable time.

## Supplementary Material

Supplementary Data
