# Supplementary material for: MEGADOCK 4.0: an ultra–high-performance protein–protein docking software for heterogeneous supercomputers
Source: Bioinformatics. 2014 Aug 6;30(22):3281–3. doi: 10.1093/bioinformatics/btu532 (PMC4221127; doi:10.1093/bioinformatics/btu532)
Supplement: Supplementary Data [file supp_30_22_3281__index.html]

MEGADOCK 4.0: an ultra–high-performance protein–protein docking software for heterogeneous supercomputers — MEGADOCK 4.0: an ultra–high-performance protein–protein docking software for heterogeneous supercomputers — MEGADOCK 4.0: an ultra–high-performance protein–protein docking software for heterogeneous supercomputers — Supplementary Data 

# MEGADOCK 4.0: an ultra–high-performance protein–protein docking software for heterogeneous supercomputers

## Supplementary Data

files

**Files in this Data Supplement:**

- Supplementary Data - docx file
