# Supplementary material for: MEGADOCK 4.0: an ultra–high-performance protein–protein docking software for heterogeneous supercomputers
Source: Bioinformatics. 2014 Aug 6;30(22):3281–3. doi: 10.1093/bioinformatics/btu532 (PMC4221127; doi:10.1093/bioinformatics/btu532)
Supplement: Supplementary Data [file supp_btu532_megadock4.0_suppl_revised.docx]

*Supplementary Information*

MEGADOCK 4.0: an ultra–high-performance protein–protein docking software for heterogeneous supercomputers

Masahito Ohue^1,2†^, Takehiro Shimoda^1,3†^, Shuji Suzuki^1,2,3^, Yuri Matsuzaki^3^,
Takashi Ishida^1^, and Yutaka Akiyama^1,3*^

^1^Department of Computer Science, Graduate of Information Science and Engineering, Tokyo Institute of Technology, 2-12-1 W8-76, Ookayama, Meguro-ku, Tokyo 152-8550, Japan

^2^Japan Society for the Promotion of Science (JSPS) Research Fellow

^3^Education Academy of Computational Life Sciences (ACLS), Tokyo Institute of Technology, 2-12-1 W8-93, Ookayama, Meguro-ku, Tokyo 152-8550, Japan

^†^These authors contributed equally to this work

^*^Corresponding author (akiyama@cs.titech.ac.jp)

**Contents**

**Supplementary Material 1**

Text S1: Scoring function with correlation functions on protein docking 2

**Supplementary Material 2**

Text S2: Hardware specifications of the TSUBAME 2.5 supercomputer 3

Table S1: Hardware specifications of TSUBAME 2.5 *Thin* nodes 3

**Supplementary Material 3**

Table S2: Benchmarking results (strong scaling) of 30,976 docking jobs 4

**Supplementary Material 4**

Table S3: The average-sized protein (FFT size *N* = 108) dataset described in the main text 5

**Supplementary Material 5**

Text S3: Installation of MEGADOCK 4.0 6

**References 8**

**Supplementary Material 1 — Text S1**

***Scoring function with correlation functions on protein docking***

One of the major docking methods is the 3-D grid-based docking technique with the fast Fourier transform (FFT) correlation approach [1]. In this method, also used on MEGADOCK 4.0, the protein structure is projected onto a 3-D grid, and the scoring function is calculated by discrete Fourier transform (DFT) and inverse discrete Fourier transform (IDFT) using the correlation of two discrete functions (protein grids), as follows:

$$\begin{matrix} S(\alpha,\beta,\gamma)= \sum_{l=1}^{N} \sum_{m=1}^{N} \sum_{n=1}^{N} R(l,m,n)L(l+\alpha,m+\beta,n+\gamma) \\ = \mathrm{IDFT}[\mathrm{DFT}[R(l,m,n)]^{\text{*}}\mathrm{DFT}[L(l,m,n)]] \end{matrix}$$

where *R* and *L* are the discrete function of the receptor (*R*) and ligand (*L*) proteins, respectively, (*l*, *m*, *n*) is a coordinate in the 3-D grid space, and (*α*, *β*, *γ*) is the parallel translation vector of the ligand protein. The asterisk operator ∗ indicates the complex conjugate of a complex number. To directly execute the simple convolution sums in *S*(*α*, *β*, *γ*), *O*(*N*^6^) calculations are required; however, this is reduced to *O*(*N*^3^ log *N*) using the FFT.

The discrete functions *R* and *L* usually take into account multiple effects, such as shape complementarity, electrostatic interaction, desolvation free energy, and so on (e.g. ZDOCK [2], PIPER [3], and SDOCK [4]). The total scoring function is the weighted sum of the partial scoring functions, according to the following example:

$$\begin{matrix} S_{\mathrm{total}}(\alpha,\beta,\gamma)= w_{\mathrm{shape}}S_{\mathrm{shape}}+w_{\mathrm{elec}}S_{\mathrm{elec}}+w_{\mathrm{desol}}S_{\mathrm{desol}} \\ S_{\mathrm{shape}}(\alpha,\beta,\gamma)= \mathrm{IDFT}[\mathrm{DFT}[R_{\mathrm{shape}}(l,m,n)]^{\text{*}}\mathrm{DFT}[L_{\mathrm{shape}}(l,m,n)]] \\ S_{\mathrm{elec}}(\alpha,\beta,\gamma)= \mathrm{IDFT}[\mathrm{DFT}[R_{\mathrm{elec}}(l,m,n)]^{\text{*}}\mathrm{DFT}[L_{\mathrm{elec}}(l,m,n)]] \\ S_{\mathrm{desol}}(\alpha,\beta,\gamma)= \mathrm{IDFT}[\mathrm{DFT}[R_{\mathrm{desol}}(l,m,n)]^{\text{*}}\mathrm{DFT}[L_{\mathrm{desol}}(l,m,n)] \end{matrix}$$

In this example, the total scoring function is calculated based on three correlation functions^[[1]](#footnote-1)^.

On the other hand, MEGADOCK requires only one correlation function, despite that the total scoring function take into account shape complementarity, electrostatic interaction, and desolvation free energy, like ZDOCK. We developed the original shape complementarity function (*S*_rPSC_) and desolvation free energy function (*S*_RDE_). The total scoring function of MEGADOCK is represented by these functions as follows:

where *S*_total_ consisted of only one correlation function. More details of the partial functions are described in previous reports [5, 6].

**Supplementary Material 2 — Text S2 and Table S1**

***Hardware specifications of the TSUBAME 2.5 supercomputer***

The TSUBAME 2.5 supercomputer is an advanced high-performance computing resource provided by the Global Scientific Information and Computing Center (GSIC) at the Tokyo Institute of Technology (<http://tsubame.gsic.titech.ac.jp/en>). Currently, TSUBAME 2.5 provides production services for which peak performance reaches 5.7 petaflops in double precision and 17 petaflops in single precision by aggressively exploiting the higher performance per watt. This is the machine achieving petaflop speed in Japan, and it ranks 13th in the top500 list and 8th in the green500 list announced at the International Supercomputing Conference 2014 (<http://www.top500.org> and <http://www.green500.org>, June 2014).

TSUBAME 2.5 consists of more than 1,400 compute nodes interconnected by high-bandwidth full-bisection Infiniband networks. There are three node types: *Thin*, *Medium*, and *Fat* nodes, which differ in their equipped memory capacity among, other specifications. All compute nodes share scalable storage systems that provide 7 PB of capacity. In this study, we used *Thin* nodes; hardware specifications are shown in Supplementary Table S1.

***Supplementary Table S1***

Hardware specifications of TSUBAME 2.5 *Thin* nodes

| CPU | Intel Xeon X5670 (2.93 GHz) (6 cores) × 2 |
| --- | --- |
| Memory | 54 GB |
| OS | SUSE Linux Enterprise Server 11 SP1 |
| GPU | NVIDIA Tesla K20X (GK110) × 3 |
| GPU Memory | 6 GB / GPU |
| Compiler | Intel C++ Compiler 14.0.2.144 |
| FFT library (CPU) | FFTW 3.2.2 |
| CUDA | CUDA 5.5 |
| FFT library (GPU) | cuFFT 5.5 |

**Supplementary Material 3 — Table S2**

***Supplementary Table S2***

Benchmarking results (strong scaling) of 30,976 docking jobs

| #Nodes *n* | 35 | 70 | 105 | 140 | 210 | 280 | 350 | 420 |
| --- | --- | --- | --- | --- | --- | --- | --- | --- |
| #CPU cores  #GPUs | 420  105 | 840  210 | 1,260  315 | 1,680  420 | 2,520  630 | 3,360  840 | 4,200  1,050 | 5,040  1,260 |
| Time *T_n_* (min) | 264.4 | 133.3 | 90.6 | 67.4 | 44.6 | 33.1 | 26.7 | 22.5 |
| Strong Scaling*_n_*^a^ | - | 0.991 | 0.973 | 0.981 | 0.988 | 0.997 | 0.990 | 0.980 |

^a^ Strong scaling value from 35 nodes (Strong Scaling*_n_* = (*T*_35_ / *T_n_*) / (*n* / 35)).

**Supplementary Material 4 — Table S3**

***Supplementary Table S3***

The average-sized protein (FFT size *N* = 108) dataset described in the main text. This dataset was used in the computational experiment of a million docking calculations (Section 3 in the main text). These protein structures are available in the ZLAB protein–protein docking benchmark 4.0 (<http://zlab.umassmed.edu/benchmark>) and Protein Data Bank (PDB) (<http://www.rcsb.org>).

| PDB ID:Chain | ZLAB Benchmark Code | #Residues |
| --- | --- | --- |
| 1ATN:A | 1ATN_r | 372 |
| 1AZS:AB | 1AZS_r | 353 |
| 1BGX:HL | 1BGX_r | 423 |
| 1BJ1:VW | 1BJ1_l | 189 |
| 1BUH:A | 1BUH_r | 294 |
| 1BVN:P | 1BVN_r | 495 |
| 1F34:B | 1F34_l | 127 |
| 1FC2:D | 1FC2_l | 414 |
| 1FQ1:B | 1FQ1_l | 292 |
| 1FQJ:A | 1FQJ_r | 317 |
| 1GXD:A | 1GXD_l | 182 |
| 1I9R:HL | 1I9R_r | 438 |
| 1JK9:A | 1JK9_r | 221 |
| 1K4C:C | 1K4C_l | 394 |
| 1K5D:AB | 1K5D_r | 339 |
| 1K74:DR | 1K74_l | 284 |
| 1KXP:A | 1KXP_r | 372 |
| 1N2C:EF | 1N2C_l | 575 |
| 1OC0:A | 1OC0_r | 373 |
| 1RLB:ABCD | 1RLB_r | 456 |
| 1XU1:ABD | 1XU1_r | 404 |
| 1YVB:A | 1YVB_r | 241 |
| 1ZHH:B | 1ZHH_l | 210 |
| 2A5T:B | 2A5T_l | 278 |
| 2G77:A | 2G77_r | 322 |
| 2OT3:B | 2OT3_r | 245 |
| 2Z0E:A | 2Z0E_r | 319 |

**Supplementary Material 5 — Text S3**

***Installation of MEGADOCK 4.0***

**Requirements**:

- FFTW3 - <http://www.fftw.org>
  --enable-float flag must be specified when you compile FFTW3
- OpenMPI - <http://www.open-mpi.org> (use MPI)
- CUDA Toolkit ver. ≥ 5.0 - <https://developer.nvidia.com/cuda-zone> (use GPU)
- GPU Computing SDK code samples (same version as CUDA Toolkit) -
  <https://developer.nvidia.com/cuda-zone> (use GPU)

**Installation**:

You can use MEGADOCK 4.0 on (a) *GPU cluster*, (b) *CPU cluster*, (c) *GPU single node*, and (d) *CPU single node*. Please see the appropriate instructions below.

(a) *GPU cluster* (GPU, MPI, & OpenMP hybrid parallelization)

Extract tarball contents

$ tar xzf megadock-4.0.tgz

$ cd megadock-4.0

Edit Makefile

CUDA_INSTALL_PATH ?= your/cuda/toolkit/install/path

CUDA_SAMPLES_PATH ?= your/cuda/sdk/install/path

FFTW_INSTALL_PATH ?= your/fftw/library/install/path

CPPCOMPILER ?= icpc, g++ or others

MPICOMPILER ?= mpicxx or others

OPTIMIZATION ?= -O3

OMPFLAG ?= -openmp (intel) or -fopenmp (g++)

Compile

$ make

A binary file megadock-gpu-dp will be generated.

(b) *CPU cluster* (MPI & OpenMP hybrid parallelization)

Extract tarball contents

$ tar xzf megadock-4.0.tgz

$ cd megadock-4.0

Edit Makefile

FFTW_INSTALL_PATH ?= your/fftw/library/install/path

CPPCOMPILER ?= icpc, g++ or others

MPICOMPILER ?= mpicxx or others

OPTIMIZATION ?= -O3

OMPFLAG ?= -openmp (intel) or -fopenmp (g++)

USE_GPU := 0

Compile

$ make

A binary file megadock-dp will be generated.

(c) *GPU single node* (GPU parallelization)

Extract tarball contents

$ tar xzf megadock-4.0.tgz

$ cd megadock-4.0

Edit Makefile

CUDA_INSTALL_PATH ?= your/cuda/toolkit/install/path

CUDA_SAMPLES_PATH ?= your/cuda/sdk/install/path

FFTW_INSTALL_PATH ?= your/fftw/library/install/path

CPPCOMPILER ?= icpc, g++ or others

OPTIMIZATION ?= -O3

OMPFLAG ?= -openmp (intel) or -fopenmp (g++)

USE_MPI := 0

Compile

$ make

A binary file megadock-gpu will be generated.

(d) *CPU single node* (OpenMP thread parallelization)

Extract tarball contents

$ tar xzf megadock-4.0.tgz

$ cd megadock-4.0

Edit Makefile

FFTW_INSTALL_PATH ?= your/fftw/library/install/path

CPPCOMPILER ?= icpc, g++ or others

OPTIMIZATION ?= -O3

OMPFLAG ?= -openmp (intel) or -fopenmp (g++)

USE_MPI := 0

USE_GPU := 0

Compile

$ make

A binary file megadock will be generated.

**References**

1. Katchalski-Katzir E, Shariv I, Eisenstein M, Friesem AA, Aflalo C and Vakser IA (1992). Molecular surface recognition: determination of geometric fit between proteins and their ligands by correlation techniques. *Proceedings of the National Academy of Sciences of the United States of America*, **89**(6): 2195–2199.
2. Mintseris J, Pierce B, Wiehe K, Anderson R, Chen R and Weng Z (2007). Integrating statistical pair potentials into protein complex prediction. *Proteins*, **69**(3): 511–520.
3. Kozakov D, Brenke R, Comeau SR and Vajda S (2006). PIPER: an FFT-based protein docking program with pairwise potentials. *Proteins*, **65**(2): 392–406.
4. Zhang C and Lai L (2011). SDOCK: a global protein-protein docking program using stepwise force-field potentials. *Journal of Computational Chemistry*, **32**(12): 2598–2612.
5. Ohue M, Matsuzaki Y, Ishida T and Akiyama Y (2012). Improvement of the protein–protein docking prediction by introducing a simple hydrophobic interaction model: an application to interaction pathway analysis. *Lecture Notes in Computer Science*, **7632**: 178–187.
6. Ohue M (2014). Protein–protein interaction network prediction based on tertiary structure data. Ph.D. thesis, Department of Computer Science, Tokyo Institute of Technology.

1. In actuality, the desolvation free energy function *S*_desol_ also often comprises multiple correlation functions, e.g., ZDOCK uses six correlation functions and PIPER uses nine correlation functions for the calculation of *S*_desol_. [↑](#footnote-ref-1)
